# Trauma and displacement in Ukraine: the challenge to medicine and politics

**DOI:** 10.1093/qjmed/hcac090

**Published:** 2022-04-05

**Authors:** B D Kelly

**Affiliations:** From the Department of Psychiatry, Trinity College Dublin, Trinity Centre for Health Sciences, Tallaght University Hospital, Dublin 24, Tallaght D24 NR0A, Ireland

On 21 March 2022, the United Nations (UN) reported that since the invasion of Ukraine on 24 February, over 10 million people were forced from their homes in order to find safety and security.^1^ This amounts to almost one-quarter of the population of Ukraine. It includes an estimated 6.5 million men, women and children who are internally displaced, and almost 3.5 million who crossed the border out of Ukraine as refugees. The most pressing needs of this enormous number of people are medicines, health services and financial resources, according to the UN.

Global media have portrayed the extraordinary courage and resilience of the Ukrainian people at this time of crisis for their country. The psychological impacts of these events are less visible in most media, but they are extensive, especially among children.^2^ Loss of control is a particular issue, undermining the degree to which people feel empowered to look after themselves and others into the future.

Previous refugee crises provide clear evidence of the social, emotional and psychological risks involved in these situations both in the short term and over the years ahead.^3^ One systematic literature review of the long-term mental health of 16 010 war refugees identified significant heterogenicity in rates of depression, post-traumatic stress disorder (PTSD) and anxiety disorder, but prevalence estimates were typically in the range of 20% and above, with some estimates reaching 88% (for anxiety disorder).^4^ These mental health conditions were most consistently associated with greater exposure to pre-migration traumatic experiences and post-migration stress, with poor post-migration socio-economic status especially associated with depression.

Ongoing conflict in Ukraine already had a significant impact on mental health prior to the 2022 invasion. In 2019, Fel and colleagues^5^ studied post-traumatic stress among Ukrainian civilians living in towns in Donbas, an eastern region of Ukraine where an armed conflict has been ongoing since 2014, and among people displaced from Donbas temporarily living in central or western parts of Ukraine. They found PTSD in 37.3% of their sample. Predictors of post-traumatic stress included loss of a loved one, living in a village rather than a city, female gender, lower educational attainment and not having health insurance.

Following the current invasion, there is a pressing need to identify what can be done to prevent and minimize the adverse psychological and psychiatric effects of the humanitarian disaster and mass displacement currently occurring in Ukraine.

The Inter-Agency Standing Committee (IASC), the longest-standing and highest-level humanitarian coordination forum of the UN system, provides important guidance for this task. The IASC brings together the executive heads of 18 organizations to formulate policy, set strategic priorities and mobilize resources in response to humanitarian crises. In 2007, the IASC published the *IASC Guidelines on Mental Health and Psychosocial Support in Emergency Settings*.^6^

These guidelines highlight the extent of the issues involved in these situations:

The psychological and social impacts of emergencies may be acute in the short term, but they can also undermine the long-term mental health and psychosocial well-being of the affected population. These impacts may threaten peace, human rights and development. One of the priorities in emergencies is thus to protect and improve people’s mental health and psychosocial well-being. Achieving this priority requires coordinated action among all government and non-government humanitarian actors. (p. 1)

The IASC guidelines present a four-level ‘intervention pyramid for mental health and psychosocial support in emergencies’, starting with ‘basic services and security’, followed by ‘community and family supports’, ‘focussed, non-specialised supports’ and, finally, ‘specialised services’, including ‘psychological or psychiatric supports’ (p. 13). Although these ‘specialised services are needed only for a small percentage of the population, in most large emergencies this group amounts to thousands of individuals’. Given the numbers involved in the current Ukrainian emergency, these specialized services are likely to be required by many.

In addition to the psychosocial interventions in the IASC guidelines, specific therapies for consideration include trauma-focused psychological interventions that have been proven to be effective for managing mental health problems and comorbidities in people exposed to complex trauma.^7^ Multicomponent interventions are the most effective treatment packages for PTSD in complex trauma and, like all interventions, should take account of cultural factors during assessment, delivery and follow-up.

These issues are important not only for people living in, and displaced from, Ukraine, but also for Russian combatants, family members and civilians. Trauma and bereavement are experienced by all sides in every conflict, as well as those caught in the middle.

The current situation in Ukraine presents a challenge to mental health services, a challenge to medicine and a challenge to all governments that seek to advance population health through conflict resolution. Rudolf Virchow, the 19th-century German pathologist, anthropologist and politician, argued that ‘medicine is a social science, and politics nothing but medicine on a large scale’.^8^

Medicine and politics both matter deeply in Ukraine today. The first and best way to minimize psychological harm is for the conflict to stop and rebuilding to begin. We need both medicine and politics to allow those affected by this conflict to start the process of grieving, healing and recovery from this humanitarian disaster.

*Conflict of interest*: None declared.
